# The Effect of Trehalose Coating for Magnetite Nanoparticles on Stability of Egg White Lysozyme

**DOI:** 10.3390/ijms23179657

**Published:** 2022-08-25

**Authors:** Asma Lajmorak, Seyyed Ali Seyyed Ebrahimi, Fatemeh Yazdian, Zahra Lalegani, Bejan Hamawandi

**Affiliations:** 1 Advanced Magnetic Materials Research Center, School of Metallurgy and Materials, College of Engineering, University of Tehran, Tehran 11155-4563, Iran; 2 Department of Life Science Engineering, Faculty of New Science and Technologies, University of Tehran, Tehran 14179-35840, Iran; 3 Department of Applied Physics, KTH Royal Institute of Technology, SE-106 91 Stockholm, Sweden

**Keywords:** magnetic nanoparticles, Fe_3_O_4_, trehalose, protein corona, lysozyme

## Abstract

In this study, the protein stability of hen egg-white lysozymes (HEWL) by Fe_3_O_4_ and Fe_3_O_4_-coated trehalose (Fe_3_O_4_@Tre) magnetic nanoparticles (NPs) is investigated. For this purpose, the co-precipitation method was used to synthesize magnetic NPs. The synthesized NPs were characterized by XRD, FT-IR spectroscopy, FE-SEM, and VSM analysis. In addition, the stability of HEWLs exposed to different NP concentrations in the range of 0.001–0.1 mg mL^−1^ was investigated by circular dichroism (CD) spectroscopy, fluorescence, and UV-Vis analysis. Based on the results, in the NP concentration range of 0.001–0.04 mg mL^−1^ the protein structure is more stable, and this range was identified as the range of kosmotropic concentration. The helicity was measured at two concentration points of 0.02 and 0.1 mg mL^−1^. According to the results, the α-helix at 0.02 mg mL^−1^ of Fe_3_O_4_ and Fe_3_O_4_@Tre was increased from 35.5% for native protein to 37.7% and 38.7%, respectively. The helicity decreased to 36.1% and 37.4%, respectively, with increasing the concentration of Fe_3_O_4_ and Fe_3_O_4_@Tre to 0.1 mg mL^−1^. The formation of hydrated water shells around protein molecules occurred by using Fe_3_O_4_@Tre NPs. Hence, it can be concluded that the trehalose as a functional group along with magnetic NPs can improve the stability of proteins in biological environments.

## 1. Introduction

Nowadays, proteins have a wide range of applications in the biomedicine field, and they are used in the production of pharmaceuticals, peptide-based therapeutics, and the treatment of diseases [1,2]. However, the formation of protein aggregates during folding and reaching the fourth structure of the proteins are some of the important issues in degenerative diseases, such as Parkinson’s, Alzheimer’s, Huntington’s, and systemic diseases such as type-II diabetes [3,4]. Issues such as protein misfolding and unfolding during the formation of protein aggregates can be due to a conversion of soluble pre-fibrils to insoluble fibrils, so it can decrease the amount of α-helicals and cause pathogenic protein aggregation [5,6]. In addition, this problem is often seen in the production of recombinant proteins in the pharmaceutical industry [7]. So, this can lead to wasted primary resources and high costs in the production of recombinant drugs [8].

Protein stability is an important issue in preventing the formation of protein aggregations in systemic diseases and in the production of protein-based recombinant drugs [9]. Although there are a number of methods such as dilution and dialysis for the recovery of aggregated proteins, most of them are not 100% efficient and cannot be used at concentrations higher than 0.1 mg mL^−1^ [10,11]. Factors such as the interactions between proteins [12], changes in the hydrophobicity and hydrophilicity of the surface of the protein, and physical and chemical [13] properties of the surface cause protein aggregation and the instability of the protein structure. However, the mechanism of these changes is not yet well understood. So, finding an effective mechanism providing the ability to increase protein stability can be helpful.

In recent years, NPs have been used for different purposes, including enzyme and protein immobilization, biosensors, biological isolation, bioassays, cell tracking, localization in hyperthermia, drug delivery, and MRIs [14,15,16]. Among the NPs, magnetic NPs (Fe_3_O_4_) have received much attention due to their properties such as nontoxicity, good biocompatibility, and super-paramagnetic properties. Despite all the positive features of super-paramagnetic NPs, the stabilization of capped magnetic NP_S_ against demolition during or after synthesis and protein denaturation during the interaction with NPs are challenges for using them in biological applications. For instance, the adsorption and binding of proteins on the NP’s surface can increase the local protein concentration and enhance the aggregation kinetics; hence, the size, surface, and chemical nature of NPs can affect the properties of NPs when they interact with proteins [17,18,19]. The use of a decorative material along with NPs can both increase protein stability and prevent the direct contact of the protein with the surface of the NPs [20,21].

One of the most common strategies to solve this problem is to coat the NPs with a biocompatible material that can stabilize the magnetic NPs structure and prevent the NPs from interacting directly with the protein [22]. On the other hand, there is the ability to combine magnetic NPs with other particles before modifying the surface and coating it with organic and inorganic agents for biological applications in order to obtain the highest efficiency. Studies have shown that the presence of magnetic Fe_3_O_4_ NPs together with gold particles leads to the formation of magnetic gold NPs (MNP-Au), and surface modification by antibodies is as a suitable method for the detection of gp51 antigen in bovine leukemia virus [23]. Magnetic NPs can be coated with a variety of inorganic or organic coatings, including surfactants, polymers, osmolytes, and amino acids, or a combination of both. There are various substances that reduce protein aggregation and can play a positive role in protein stability, such as prolin, glutamine, sucrose, glucose, arginine, chitosan, and trehalose [24,25,26,27,28,29,30,31]. Among them, trehalose was selected as the coating material for Fe_3_O_4_ NPs. Osmolytes, such as trehalose, have excellent power to stabilize the protein’s structure when subjected to dehydrated conditions or chemical or thermal stresses [32]. They help proteins change their structure from disordered polypeptides to the native state and prevent the formation of harmful aggregation and misfolding [33]. They are resistant to all kinds of environmental stresses, sub-zero temperatures, and heat shocks [34,35,36].

The interaction between proteins and NPs results in the formation of a biological structure known as the corona protein. With regard to this issue, some research has been conducted on the formation of protein corona during the interaction of proteins and various NPs, such as silver [37], gold [38], Fe_3_O_4_ [39], and silica [40]. In other words, water molecules (surface and bulk water molecules) around the protein play an important role in the biological activity, folding/unfolding, and stability/instability of the proteins’ structure and their interactions with the environment; so, by strengthening the hydration layer, the stability of the protein can be changed. Therefore, when magnetic NPs and HEWL are in contact with each other, a protein corona will form on the surface of the NPs. In the meantime, a harder and more stable corona is formed with greater affinity, which leads to the formation of a denser hydration layer around the protein [41,42,43,44,45]. However, highly soluble kosmotropic solvents stabilize native protein structures by burying hydrophobic residues while in the presence of chaotropic co-solvents. Hydration layer atoms are disrupted by this, which leads to protein destabilization [46,47]. This makes it necessary to study the function of proteins in aqueous media in the presence of NPs with different operators to achieve maximum stability. Therefore, the stability and function of proteins when interacting with other molecules is a major challenge, and if the appropriate environment and NPs are not selected, it can have the opposite effect and lead to instability and the formation of protein aggregates.

In this work, Fe_3_O_4_ and Fe_3_O_4_@Tre magnetic NPs were used to investigate the effect of these NPs on stability, second structure, and function of HEWL, and a comparative study has been performed between them. Lysozyme is a ubiquitous hydrolytic enzyme having antibacterial activity. This enzyme acts as a defense mechanism by lysing the bacteria. It can also be used to deactivate bacterial cells in the presence of antimicrobial peptides, and therefore, it participates in the chain of biochemical and immunological reactions [48,49]. This bioactive macromolecule is present in many living organisms: breast milk, intestinal mucus, saliva, urine, and cerebrospinal fluid in different concentrations. In addition, it has wide applications in the preservation of food products, clinical diagnosis of diseases, antineoplastic activities, and in anti-inflammatory drugs [50,51]. The reason for using lysozyme as a model protein is its biophysical properties, such as its three-dimensional structure, folding–unfolding mechanism, as well as its well-understood conformational stability information [52,53]. In this study, we have used lysozyme as a small cationic protein due to its high purity, solubility, low cost, and its high homologous similarity with the human lysozyme. On the other hand, the presence of positive charge at the physiological pH makes the lysozyme a suitable choice for adsorption on charged nanoparticles, especially magnetite NPs [54]. Consequently, we tried to find a suitable concentration range of decorated NPs to increase the stability of the structure. Understanding the impact of NPs on protein structure and function in comparison with native proteins is effective in the applications of recombinant drug production and in the recovery of protein aggregates to their original forms.

## 2. Results and Discussion

### 2.1. Characterization of Magnetic NPs

**Figure 1:**
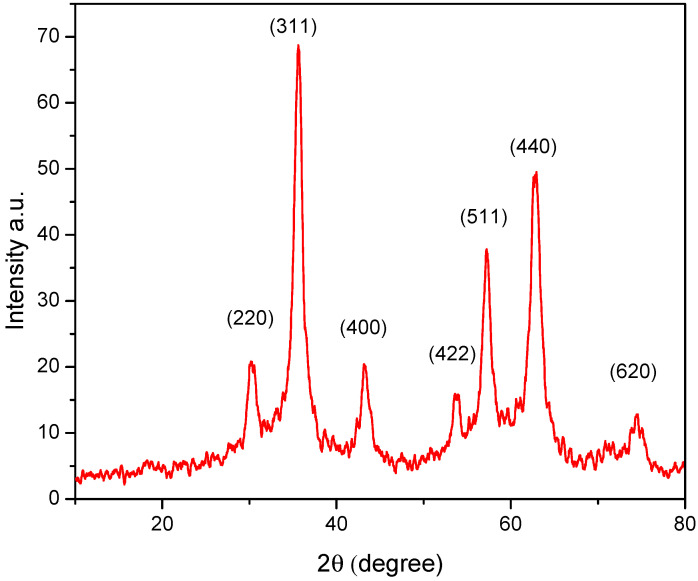
XRD pattern of Fe_3_O_4_@Tre NPs.

Figure 1 shows the XRD pattern that was performed to investigate the crystal structure, grain size, and composition of the magnetic NPs. According to the XRD pattern, the material is well crystallized. It represents the formation of Fe_3_O_4_ NPs with a spinal structure (JCPDS Card No: 00-003-0863). Using the Debye–Scherrer equation, the size of Fe_3_O_4_ and Fe_3_O_4_@Tre NPs were calculated as 44.3 nm and 38.5 nm, respectively.

**Figure 2:**
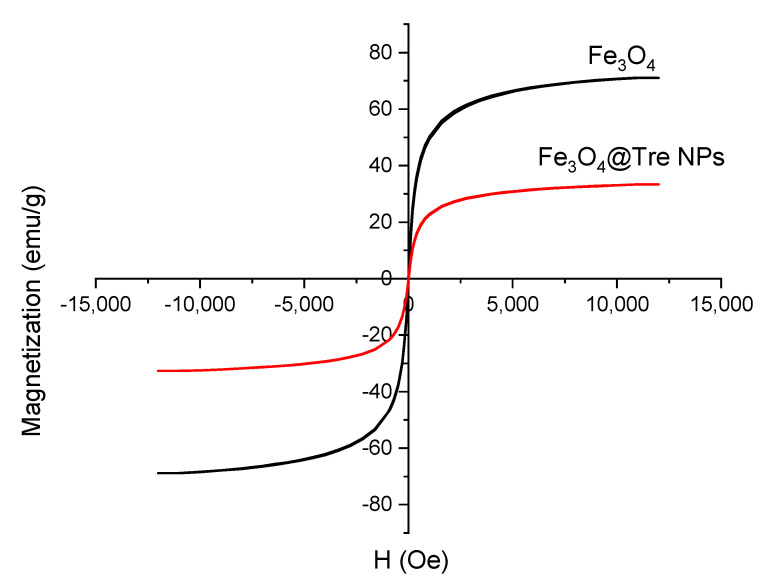
VSM diagrams of Fe_3_O_4_ and Fe_3_O_4_@Tre NPs.

Figure 2 represents the VSM results of magnetic NPs. According to Figure 2, the magnetization properties of Fe_3_O_4_ NPs have decreased after using the Tre. Also according to Figure 2, the saturation magnetization values for Fe_3_O_4_ and Fe_3_O_4_@Tre NPs were 76.1 and 37.3 emu g^−1^, respectively. Additionally, Fe_3_O_4_@Tre NPs had lower M_s_ in comparison with Fe_3_O_4_ NPs. The lower M_s_ of Fe_3_O_4_@Tre NPs is due to the diamagnetic property of Tre, which quenches the magnetic moment of Fe_3_O_4_ NPs [55]. 

**Figure 3:**
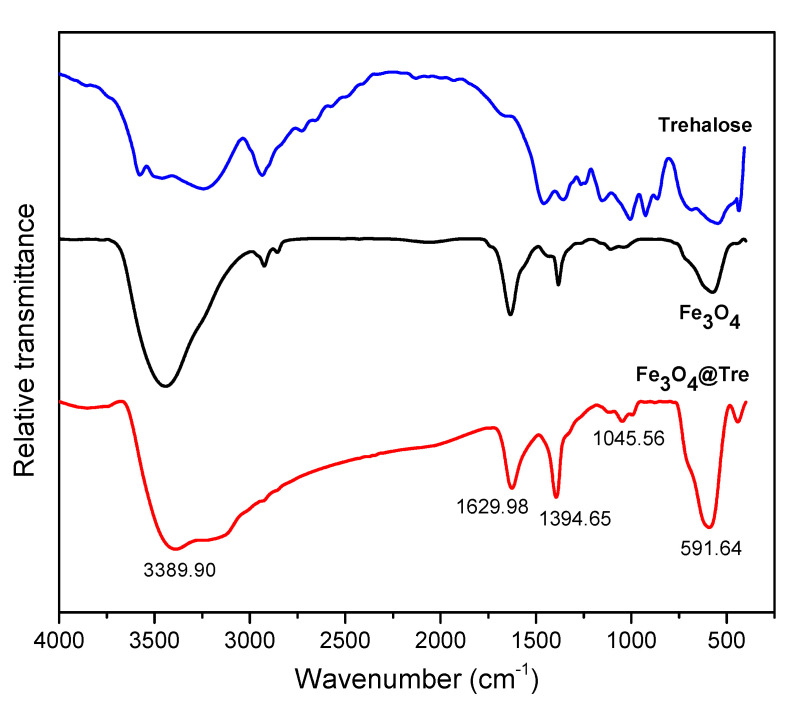
FT-IR spectra of trehalose, bare Fe_3_O_4_ NPs, and Fe_3_O_4_@Tre NPs.

Figure 3 shows the FT-IR spectra of the Fe_3_O_4_ and Fe_3_O_4_@Tre NPs. The peak at about 591 cm^−1^ is the characteristic absorption of the Fe–O band. In fact, this peak indicates the presence of magnetic particles. The peaks in the 1000–1390 cm^−1^ region are attributed to the C–O vibrations. The broad peak at about 3500 cm^−1^ is related to the stretching vibrations of hydroxyl groups (O–H), which indicates the formation of polysaccharide shells around the NPs [56]. In addition, the peaks at about 1395 cm^−1^ and 1630 cm^−1^ attribute to amine groups (NH_2_) of Tre. According to the results of the FT-IR test, the Tre was successfully decorated on the surface of magnetic NPs.

The DLS test was used to obtain the zeta potential and hydrodynamic size of the Fe_3_O_4_ and Fe_3_O_4_@Tre NPs in 100 mM potassium phosphate buffer (PBS) with pH = 7.4. The results of the DLS test are listed in Table 1.

According to the zeta potential data, both NPs have good colloidal stability (<−30). The negative charge of the Fe_3_O_4_@Tre NPs leads to a decrease in the number of destructive interactions between proteins and NPs that can affect protein stability. In addition, the negative charge of the NPs interacts with the positive charge of the water molecules around the protein, causing the protein and NPs to bind to each other.

**Figure 4:**
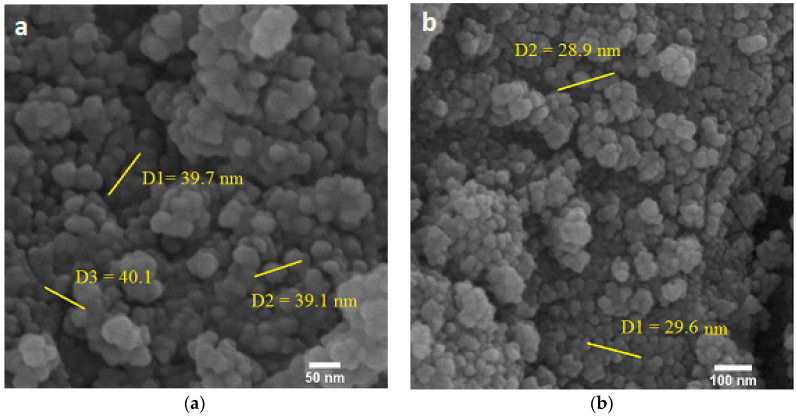
FE-SEM images of (**a**) bare Fe_3_O_4_ NPs, and (**b**) Fe_3_O_4_@Tre NPs.

Figure 4 shows the FE-SEM images of Fe_3_O_4_ and Fe_3_O_4_@Tre NPs. The spherical morphology of the NPs is obvious from the FE-SEM images. The particle sizes of the Fe_3_O_4_ and Fe_3_O_4_@Tre NPs were obtained and are about 43 and 34 nm, respectively, which is in good agreement with the results obtained from the DLS test (Table 1).

### 2.2. Fluorescence Measurement

**Figure 5:**
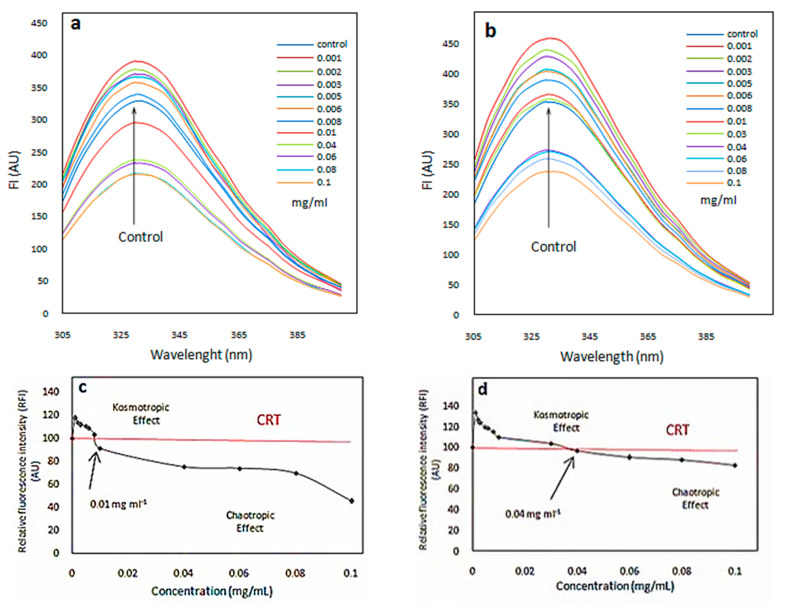
(**a**,**b**) Diagrams of tryptophan fluorescence in the presence of different concentrations of bare Fe_3_O_4_ and Fe_3_O_4_@Tre NPs, respectively; (**c**,**d**) diagrams of the relative fluorescence intensity showing a dual concentration effect of Fe_3_O_4_ and Fe_3_O_4_@Tre NPs compared to the control state (absence of NPs), respectively.

According to the literature [57], the fluorescence measurement was performed to evaluate the microenvironment nature of amino acids in the protein. For this purpose, the intrinsic fluorescence intensity of tryptophan was 62 and 108 in HEWL with strong fluorophore being used in the presence and absence of NPs, respectively. The molecular weight of HEWL is 14.7 KDa. Therefore, the small size of the molecules and their positive charge cause the aggregation of the protein on the surface of the negatively charged magnetic NPs [58]. The large surface area of NPs, the formation of a protein corona structure, and the interaction of protein molecules with water molecules at the surface of the NPs can create a kosmotropic effect [44,59]. There is a concentration range of NPs which can have either stabilizing (kosmotropic) or degrading (chaotropic) effects. Figure 5 shows the fluorescence intensity graphs. The intensity of the fluorescence emission is determined by the environment of the fluorophores in the model protein [60]. Figure 5a,b show the fluorescence intensity changes at different wavelengths for Fe_3_O_4_ and Fe_3_O_4_@Tre NPs, respectively. The concentration range of NPs is 0.001–0.1 mg mL^−1^. According to the graphs of Figure 5a,b, there is a dual effect on the tertiary structure of the lysozyme protein. In addition, it can be seen that at a specific concentration range both NPs can improve the stability of the structure, and at the concentration ratio of threshold (CRT), the structure is destroyed.

According to Figure 5a, the presence of Fe_3_O_4_ NPs increased the fluorescence intensity in the concentration range of 0.001–0.1 mg mL^−1^. In other words, this concentration range of Fe_3_O_4_ NPs stabilizes the protein structure, while, according to Figure 5b, Fe_3_O_4_@Tre NPs improved the protein stability over a wider concentration range of 0.001–0.04 mg mL^−1^. Hence, it had a better effect on the tertiary structure of the protein. In addition, according to Figure 5b, Fe_3_O_4_@Tre NPs bind more strongly to proteins. The CRTs for the Fe_3_O_4_ and Fe_3_O_4_@Tre NPs in a constant concentration of HEWL (0.2 mg mL^−1^) were estimated to be 0.0084 and 0.01824 mg mL^−1^, respectively.

Figure 5c,d are derived from the graphs of Figure 5a,b and indicate the presence of a dual concentration effect. It can be seen that there is a chaotropic effect at higher concentrations (>CRT) and a kosmotropic effect at lower concentrations (<CRT). In addition, according to Figure 5c,d, the Fe_3_O_4_@Tre NPs are better stabilizers for the protein structure, and they can be a structure-maker in HEWL. The presence of Tre around protein molecules prevents direct protein–protein interactions and stabilizes the colloidal stability of the protein [12]. However, at concentrations higher than CRT, the polarity of the environment around the protein is altered. It causes an opening in the structure and reduces the stability of the protein.

### 2.3. Circular Dichroism Spectroscopy

**Figure 6:**
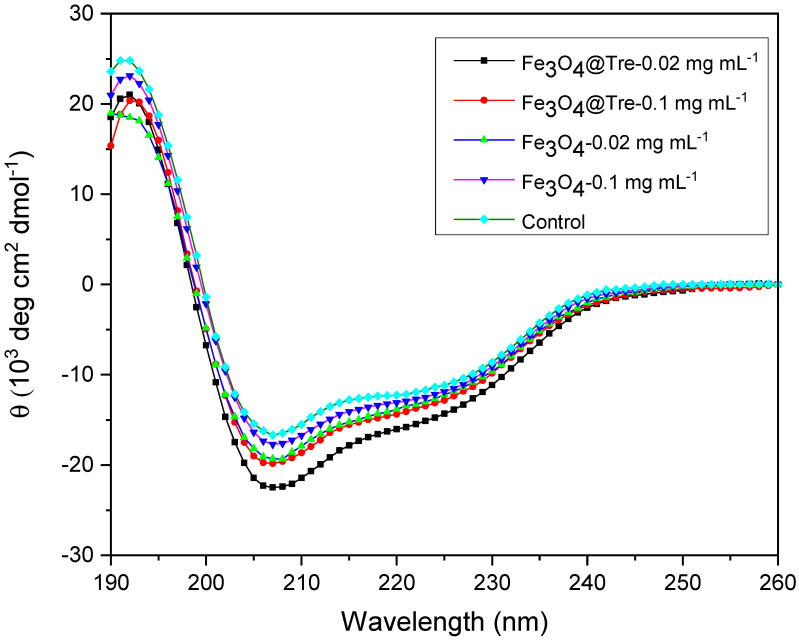
CD spectra of 0.2 mg mL^−1^ lysozyme in the presence of Fe_3_O_4_ and Fe_3_O_4_@Tre NPs with different concentrations.

CD spectroscopy was used to investigate the α-helix and β-sheets changes in the protein structure. According to the fluorescence test, the concentrations of 0.02 mg mL^−1^ and 0.1 mg mL^−1^ (for both Fe_3_O_4_ and Fe_3_O_4_@Tre NPs) were selected to use for the CD test. Figure 6 shows the resulting CD spectra (elliptic state vs. wavelength). As can be seen, the amount of helicity in the protein structure is increased in the presence of Fe_3_O_4_@Tre NPs compared to the bare Fe_3_O_4_ NPs. In addition, the elliptic state is decreased at wavelengths of 208–240 nm. This wavelength range corresponds to the environment of hydrogen bonds in the secondary structure of the HEWL.

**Figure 7:**
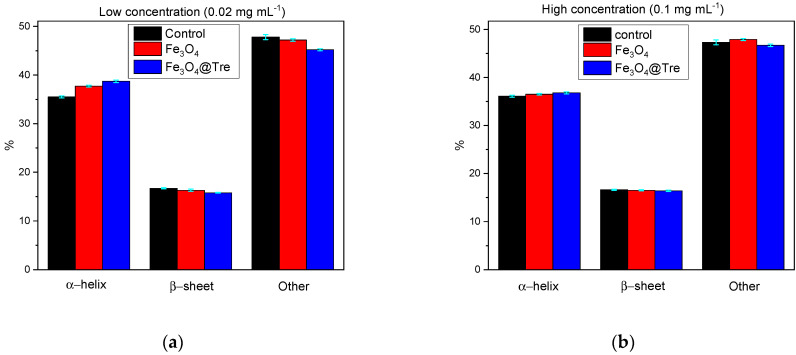
Qualitative diagrams of CD test results in different concentrations of (**a**) 0.02 mg mL^−1^ NPs, and (**b**) 0.1 mg mL^−1^ NPs.

Figure 7 shows the qualitative diagrams derived from CD test data. As can be seen, the amount of α-helix at the concentration of 0.02 mg mL^−1^ was increased to 37.7% and 38.7% for Fe_3_O_4_ and Fe_3_O_4_@Tre NPs, respectively, in comparison with 35.5% helicity in the native protein. Increasing the concentration of NPs to 0.1 mg mL^−1^, the helicity reaches 36.1% and 36.4% for Fe_3_O_4_ and Fe_3_O_4_@Tre NPs, respectively.

As a kosmotrope agent, Fe_3_O_4_@Tre NPs can stabilize the enzyme structure by making a hydration layer around the enzyme. The active OH groups of Tre make a suitable hydrophilic environment around the protein. It can prevent the formation of insoluble fibrils and β-sheets [61,62,63]. Tre causes protein stability and increases the α-helix by creating hydration sites and increasing the viscosity and surface tension.

### 2.4. UV-Visible Investigation of Lysozyme Activity Limit

**Figure 8:**
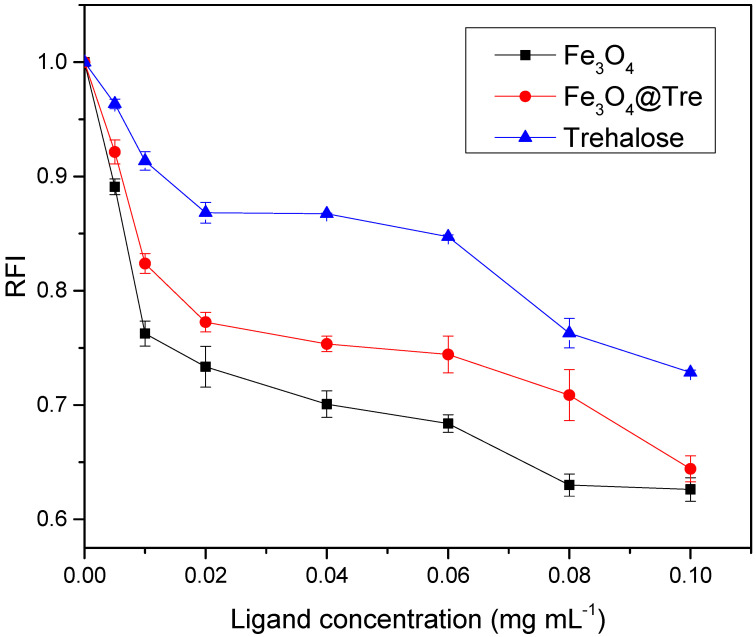
UV-Vis test evaluation of protein activity.

Lysozymes can lysis the cell wall of the bacteria, especially the gram-positive bacteria. Figure 8 shows the UV-Vis investigation of lysozyme activity. It shows the interaction between *M. luteus* and lysozyme in the presence and absence of NPs. According to Figure 8, it can be concluded that bacterial activity decreases by increasing the concentration of NPs. It can also be seen that the graph of Fe_3_O_4_@Tre NPs is higher than that of the bare Fe_3_O_4_ NPs, which means that Fe_3_O_4_ NPs, without functionalizing with Tre, have a more negative effect on the limitation of bacterial activity. This is additional evidence that the presence of Tre as a kosmotrope agent along with magnetic Fe_3_O_4_ NPs is able to further stabilize the protein structure. The data obtained from this test are consistent with the fluorescence results.

### 2.5. Protein Corona Formation

**Figure 9:**
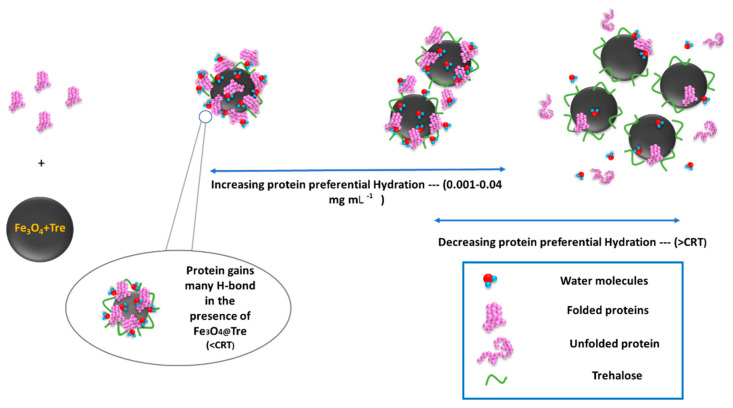
Schematic of protein change according to the NPs concentrations.

The protein corona, which is the result of the interaction between NPs and proteins, can cause the biological distribution and toxicity of NPs. Size, geometry, solubility, and surface properties of NPs as the physicochemical parameters of NPs and environment properties, such as pH and temperature can affect the structure and composition of the corona [45,64,65]. The positive charge of proteins with an isoelectric point of 10.7 and the negative charge of magnetite NPs provide the possibility of strong connections and the formation of a corona protein. In addition, the asymmetric charge distribution on the surface of the protein, despite making the interactions between protein and NPs more complicated, still provides the possibility of NPs and proteins to bind [66,67]. Corona adsorption on magnetic NPs surfaces not only does not change the magnetic properties of NPs, but it also improves the colloidal stability and reduces the biological toxicity of NPs [68]. Choosing the right concentration of NPs and a suitable coating will result in a stronger corona and, consequently, a denser hydration layer around the protein. There are some protein–ligand studies from the past decade [28,44,61,69,70,71,72,73,74] which are listed in Supplementary Materials Table S1. According to the obtained results in this study, the kosmotropic effect can occur in a certain concentration range of 0.001–0.04 mg mL^−1^ and 0.001–0.01 mg mL^−1^ for Fe_3_O_4_@Tre and Fe_3_O_4_ NPs, respectively. Therefore, it can be concluded that at lower magnetic NP–protein ratios, the surface of the NPs can be saturated by the corona protein. However, with the increasing protein concentration, the kosmotropic effect of NPs disappears. Figure 9 shows a schematic of protein changes with increasing NPs concentration. It shows that low concentrations lead to the formation of more hydrogen bonds around the protein and hence increase the stability of the protein. According to Figure 9, the hydration pattern in the lysozyme has changed with its adsorption on the surface of the NPs in such a way that at high magnetite–protein ratios (>threshold) the formation of a protein corona is greatly reduced and the NPs are scarcely saturated by proteins. As a result, the surfaces of magnetite NPs are exposed to the solvent; hence, they interfere in protein hydration and play the role of a chaotropic agent. This is while reducing the ratio of NP–protein to a suitable concentration range (not too low); the NPs are sufficiently saturated by the protein corona. In addition to strengthening the adsorbent interactions by binding to the residues, it reduces the effect of NPs in preferential hydration and acts as a kosmotrop agent to make the protein structure more orderly. So far, improvements in the HEWL globular and bovine serum albumin (BSA) protein stability has been reported [44,59,75] in the presence of different concentrations of NPs, and the results indicate the dual concentration-dependent effect of NPs on the stability and structure of the protein. The structure-making and structure-breaking performance of NPs on HEWLs have been reported for low and high NP–protein ratios. Fattah et al. [44] reported the kosmotropic concentration range for magnetite NPs to be about 0.77 mg/μmol for BSA and 0.47 mg/μmol for HEWL. The results of their CD and UV–vis spectroscopy tests showed that the α-helix percentage of BSA is about 53.68%, 41.19%, and 34.7% for no-ligand, 0.02 mg/mL, and 0.1 mg/mL of NPs, respectively, and 26.29%, 27.92%, and 29.56% for HEWL.

## 3. Materials and Methods

### 3.1. Materials and Instrumentation

Hen egg-white lysozyme protein (HEWL, molecular weight of 14.3 KDa) as a model protein and trehalose were purchased from Sigma Aldrich (St. Louis, MO, USA). A dried bacterial cell wall (Gram-positive *Micrococcus luteus*—*M. luteus*) was used as the natural substrate of HEWL. Ferric chloride hexahydrate (FeCl_3_·6H_2_O, Merck KGaA, Darmstadt, Germany), ferrous chloride tetrahydrate (FeCl_2_·4H_2_O, Merck KGaA, Darmstadt, Germany), ammonia (NH_3_, 25 wt%), and deionized water were used to synthesize of magnetic NPs. Sodium phosphate monobasic monohydrate (NaH_2_PO_4_·H_2_O, Sigma Aldrich, USA) and sodium phosphate dibasic (N_2_HPO_4_, Sigma Aldrich, USA) were also used for the preparation of protein solutions and modified NPs.

Magnetic NPs were characterized using various analytical methods. Fourier transform infrared (FT-IR, Bruker VERTEX 70, Ettlingen, Germany) spectroscopy was used to investigate the presence of trehalose on the surface of NPs. FT-IR spectra for all the samples were recorded in the transmission mode in the range 4000–400 cm^−1^. X-ray diffraction analysis (XRD, Rigaku-Dmax 2500, Tokyo, Japan) was performed for the phase identification. The diffractometer was used under Cu-Kα single-wave radiation at a current of 40 mA and a voltage of 40 kV with a step size of 0.02 degrees and a step time of 0.25 seconds in the angular range of 0–80°. The morphology of NPs was examined by field emission scanning electron microscopy (FE-SEM, KYKY SBC-12, Beijing, China). The saturation magnetization of NPs was also characterized by vibrating sample magnetometry (VSM, Kavir Co., Kashan, Iran, 15kOe applied field). A magnetic field of 1.5 Tesla was used to extract the hysteresis loop. For this purpose, a voltage of 50 V was applied to create a current of 100 A. In addition, the stopping time at each stage of applying the magnetic field was considered to be 10 seconds. Colloidal properties of magnetic NPs in phosphate buffer saline (PBS, 100 mM, pH = 7.4) were estimated by using a dynamic light scattering (DLS, Malvern ZS-Nano series, Worcestershire, UK) instrument. 

The fluorescence emission of HEWL was recorded in the wavelength range of 300–400 nm using a spectro-fluorometer (Bio-Tek, Synergy^TM^ H4 hybrid microplate reader, equipped with GEN5 software, Winooski, VT, USA). An amount of 0.2 mg mL^−1^ of HEWL was incubated with variable concentrations of magnetic NPs (0 – 0.1 mg mL^−1^). Afterwards, the prepared samples were placed in a shaker for 4 hours. The sodium phosphate buffer (0.1 mM and pH 7.4) was used to record the fluorescence emission. Excitation slits and nominal band pass of emissions were set at 5 nm. A circular dichroism spectrometer (CD, AVIV 215, Lakewood, CA, USA) was used to determine the helicity changes. To determine the percentage of different secondary structural elements, molar residual ellipticity (MRE) was calculated using CDNN software. Each dataset was an average of three scans between 208 nm and 290 nm. In addition, UV-Vis spectrophotometer (VarianInc., equipped with Cary 100 software, Sydney, Australia) was used to determine the lysis rate of *M. luteus* and protein activity. For this purpose, 100 μL HEWL stock solution (concentration 0.2 mg mL^−1^) was added to 2.5 mL of *M. luteus* suspension in potassium phosphate buffer (0.01% *w*/*v*), and UV-Vis absorption changes were recorded at 450 nm.

### 3.2. Synthesis of Fe_3_O_4_ and Fe_3_O_4_@Tre NPs

Fe_3_O_4_ and Fe_3_O_4_@Tre NPs were synthesized by chemical coprecipitation method according to the following procedure. First, 24 mmol FeCl_3_·6H_2_O and 16 mmol of FeCl_2_·4H_2_O were dissolved in 190 mL of deionized water at room temperature while stirring under an N_2_ stream to achieve a stoichiometric ratio of 2Fe^3+^:Fe^2+^. Then, 18 mL of ammonia (25 wt%) was slowly added to the iron salt solution. Then, the solution was stirred for 30 min. Immediately, a black magnetite precipitate was formed. Subsequently, the magnetic precipitates were separated from the solution using a magnet and washed with deionized water and methanol three times. Finally, the precipitates were dried at room temperature. 

The chemical co-precipitation method was also used to synthesize Fe_3_O_4_@Tre. For this purpose, 175 mg Tre was dissolved in 5 mL deionized water and was then added to 50 mL FeCl_2_·4H_2_O (20 mM) and FeCl_3_·6H_2_O (40 mM) iron salts. Separation of precipitates from the black-colored solution was performed using an external magnet and was consequently washed with deionized water and methanol. This process was repeated six times and then dried for 1 day at room temperature.

## 4. Conclusions

In this study, the effects of the type and concentration of Fe_3_O_4_ magnetic NPs with and without trehalose on the stability of HEWL proteins in aqueous solutions were investigated. According to the results, both magnetite and magnetite-trehalose NPs with particle sizes of 41 and 32 nm, respectively, have good colloidal stability (<−30), and both magnetic NPs played a constructive role at low concentrations by showing a kosmotropic effect via increasing the stability of the protein structure. When passing the CRT (0.0084 and 0.01824 mg mL^−1^ for Fe_3_O_4_ NPs and Fe_3_O_4_@Tre, respectively), the chaotropic effect appeared, and NPs lost their protective effect. According to the fluorescence analysis, below the CRT (0.01 and 0.04 mg mL^−1^ for Fe_3_O_4_ and Fe_3_O_4_@Tre, respectively) both NPs exhibit structure-making behavior. However, with a greater increase in the concentration of NPs, this effect gradually decreased and showed a structure-breaking behavior by affecting the hydration layer around the protein. Interestingly, the presence of trehalose as a carbohydrate along with magnetite NPs enhanced the kosmotropic property. In this regard, a harder and more stable corona, along with a denser hydrated layer around the protein, were created, which eventually increased the stability of the HEWL. CD spectroscopy showed that the presence of Tre improves the state of the secondary structure of the lysozyme by increasing the helical content. An increase in the amount of α-helix after interaction with both MNPs was observed (37.7% and 36.1% increase in the helicity for 0.02 and 0.1 mg mL^−1^ of Fe_3_O_4_, respectively, and 38.7% and 36.4% for 0.02 and 0.1 mg mL^−1^ of Fe_3_O_4_@Tre, respectively). According to the results of this research, it can be concluded that using functional groups with NPs can improve the stability of proteins in biological environments or even reduce the formation of protein aggregates.

## Figures and Tables

**Table 1:** Zeta potential and hydrodynamic size of Fe_3_O_4_ and Fe_3_O_4_@Tre NPs in 100 mM PBS with pH = 7.4.

NPs	Size (nm)	Zeta Potential (mV)
Fe_3_O_4_	41	−32.6
Fe_3_O_4_@Tre	32	−36.1

## Data Availability

Data sharing not applicable to this article as no datasets were generated or analyzed during the current study.

## Supplementary Materials

The following supporting information can be downloaded at: https://www.mdpi.com/article/10.3390/ijms23179657/s1.
