# Supplementary material for: The Effect of Trehalose Coating for Magnetite Nanoparticles on Stability of Egg White Lysozyme
Source: Int J Mol Sci. 2022 Aug 25;23(17):9657. doi: 10.3390/ijms23179657 (PMC9456156; doi:10.3390/ijms23179657)
Supplement: Supplementary file 1 [file ijms-23-09657-s001.zip › ijms-1870352-supplementary.pdf]

Supplementary material for

The Effect of Trehalose Coating for Magnetite Nanoparticles on Stability of Egg White Lysozyme

Asma Lajmorak [1], Seyyed Ali Seyyed Ebrahimi [1,*], Fatemeh Yazdian [2], Zahra Lalegani [1] and Bejan Hamawandi [3,*]

[1] Advanced Magnetic Materials Research Center, School of Metallurgy and Materials, College of Engineering, University of Tehran, Tehran 11155-4563, Iran
[2] Department of Life Science Engineering, Faculty of New Science and Technologies, University of Tehran, Tehran 14179-35840, Iran
[3] Department of Applied Physics, KTH Royal Institute of Technology, SE-106 91 Stockholm, Sweden
* Correspondence: saseyyed@ut.ac.ir (S.A.S.E.); bejan@kth.se (B.H.)

Table S1. Protein-ligand studies during the past decade.

Ligand type	Protein type	Results	Year	Ref.
Trehalose	alpha-synuclein (AS)	CD results: trehalose at a high concentration (100mM) could slow down the formation of β-sheet aggregates, stabilize and increase the partially-folded oligomers or β-sheet-rich protofilaments, and prevent the formation of the mature A53T AS fibrils during the incubation.	2012	[61]
Tre-Ag NPs	HEWL	CD, UV, and fluorescence results: the use of different amounts of trehalose along with Ag NPs to evaluate the stability of the protein shows that creates a condition in which the NPs and the protein co-exist together without having any adverse effect on the protein structure. In the presence of trehalose (0.05M), the percentage of α-helix in HEWL was changed from 23.45% to 24.40%. And with increasing of trehalose (0.5M), α-helix was increased to 33.01%.	2015	[69]

Fe NPs	HEWL	CD results: the secondary structure of HEWL has not changed with increasing Fe NPs concentrations but with the raising amount of NPs, some changes occurred in the tertiary structure of the protein.	2016	[70]
Tre-Au NPs	HD150Q cells (huntingtin)	UV-Visible: designed NPs had 20-30 nm Au core with about 350 trehalose molecules per particle on the surface of NPs. Trehalose as a good agent with Au NPs reduced the amount of HD150Q cells aggregation.	2017	[71]
Poly(Trehalose)/ Fe_2O_3	HD150Q	CD results: Trehalose-functionalized zwitterionic NPs inhibited amyloid/ polyglutamine aggregation and decreased the stability of aggregate.	2017	[28]
Gold nanoclusters (AuNCs)	HEWL	CD, UV–vis spectroscopy: using different aqueous environments like urea, and sodium dodecyl sulphate and detecting lysozyme unfolding when they interacted with Au NCs. This study highlights the possibility of using Au NCs as a useful probe for protein unfolding applications.	2019	[72]

Prolin-AuNPs	HEWL	CD, UV–vis spectroscopy: proline along with Au NPs, acted as a hydrophobic agent to stabilize and thus reduce protein aggregates. The binding of proline at hydrophobic patches of HEWL inhibited the formation of prefibrils. The data revealed an increase in helicity for the HEWL-Pro-AuNPs system. After incubating these NPs with protein, The α-helix and β-sheet percentage was estimated 28% and 21% respectively.	2019	[73]
Fe_3O_4@PEG	BSA and HEWL (Hen egg white Lysozyme)	Two different environments were used to study the protein stability in the presence of magnetic NPs. The results of their CD, UV–Vis spectroscopy tests showed that the α-helix in BSA was estimated 53.68%, 41.19% and 34.7% for no ligand, 0.02 mg/ml, and 0.1 mg/ml NPs, respectively and 26.29%, 27.92% , and 29.56% for HEWL.	2020	[44]
Au NPs	HSA /BSA and HEWL	The presence of 0.05 mM of Au NPs led to a change in helicity of secondary structure for model proteins, the results indicated that helicity changed in HSA (from 5.74% to 6.41%), BSA (from 6.85% to 6.81%), and HEWL (from 15.53% to 21.41%).	2021	[74]
